# Systematic review and meta-analysis of belatacept versus calcineurin inhibitors on risk of post-transplant diabetes mellitus in kidney transplant recipients

**DOI:** 10.3389/fimmu.2026.1615875

**Published:** 2026-02-13

**Authors:** Xuchuan Wang, Dandan Song, Shufu Hou, Aiju Liu, Jing Gao, Lei Liu

**Affiliations:** 1 Department of Ophthalmology, Shandong Provincial Third Hospital,Shandong University, Jinan, China; 2 Department of Neurology, Shandong Provincial Third Hospital, Shandong University, Jinan, China; 3 Central Hospital Affiliated to Shandong First Medical University, Jinan, Shandong, China; 4 Department of Labor Union Office, Shandong Provincial Third Hospital, Shandong University, Jinan, China

**Keywords:** belatacept, calcineurin inhibitors, immunosuppressive agents, kidney transplantation, new-onset diabetes

## Abstract

**Background:**

The novel immunosuppressant belatacept demonstrates a unique mechanism of action that significantly improves renal function and reduces metabolic complications. However, systematic evidence comparing the risk of post-transplant diabetes mellitus (PTDM) and overall safety profiles between belatacept-based regimens and calcineurin inhibitor (CNI)-based protocols remains limited. This meta-analysis aims to synthesize high-quality evidence to determine the comparative efficacy of these two regimens in PTDM prevention and safety outcomes, thereby providing robust guidance for clinical decision-making.

**Methods:**

We systematically searched PubMed, Cochrane Library, CNKI, and EMBASE for studies published until November 30, 2024, comparing belatacept versus calcineurin inhibitors (CNIs) regarding PTDM risk in kidney transplant recipients. The primary outcome was PTDM incidence. Following data extraction and quality assessment, we performed pairwise meta-analyses to compare PTDM risk between belatacept (either less intensive(LI) or more intensive (MI) regimen) and CNIs. Bayesian network meta-analysis (WinBUGS 1.4.3) was then conducted for indirect comparison between belatacept LI and MI regimens.

**Results:**

The initial search yielded 3,206 records. After deduplication, title/abstract screening, and full-text evaluation, 6 studies involving 1,737 kidney transplant recipients were included in the final analysis. Compared with CNIs, belatacept demonstrated significant reductions in PTDM risk for both the LI (RR = 0.65, 95% CI 0.52-0.81, p<0.001; I²=30%) and MI (RR = 0.65, 95% CI 0.52-0.81, p<0.001; I²=30%) regimens. Bayesian network meta-analysis revealed no statistically significant difference between the LI and MI regimens.

**Conclusions:**

This meta-analysis demonstrates that belatacept significantly reduces PTDM risk compared to CNIs, a finding consistent with previous studies. Notably, both LI and MI dosing regimens showed protective effects, suggesting that even low-intensity belatacept therapy could serve as a viable alternative to CNIs, particularly for patients requiring reduced immunosuppressive toxicity.

**Systematic review registration:**

https://inplasy.com/, identifier INPLASY202540041.

## 1 Introduction

Kidney transplantation, as the preferred treatment for end-stage renal disease, significantly improves patient survival and quality of life. However, the metabolic complications associated with long-term immunosuppressive therapy remain a major clinical challenge. Among these, PTDM represents a clinically significant risk factor for graft loss and patient mortality, alongside other major determinants including cardiovascular disease, infections, and malignancies (1, 2). Studies indicate that PTDM is associated with an increased risk of cardiovascular events, higher infection rates, and accelerated decline in graft function, leading to a 3.67-fold increase in 5-year mortality (3, 4). CNIs, such as tacrolimus and cyclosporine, are considered central drivers of PTDM due to their direct β-cell toxicity and insulin resistance-inducing effects (5, 6). In contrast, belatacept, a selective T-cell costimulation blocker, has emerged as a potential alternative to CNIs, offering a more favorable metabolic profile. However, its efficacy in reducing PTDM risk remains controversial (7, 8).

Belatacept inhibits T-cell activation by blocking the CD28/B7 costimulatory pathway, thereby avoiding the direct pancreatic β-cell damage and metabolic disturbances caused by CNIs (9). Preclinical studies suggest that belatacept improves insulin sensitivity and reduces pro-inflammatory cytokine secretion (e.g., IL-6, TNF-α) from adipose tissue, potentially lowering PTDM risk (10–12). A randomized controlled trial (RCT) of 1,209 kidney transplant recipients demonstrated that belatacept-treated patients had a lower incidence of PTDM and improved glycated hemoglobin (HbA1c) levels compared to cyclosporine-treated patients (13). However, some cohort studies found no significant difference in PTDM incidence between belatacept and CNIs (14), suggesting that its protective effects may depend on baseline characteristics (e.g., obesity, hepatitis C infection) or concomitant immunosuppressive regimens (15). These inconsistencies may stem from heterogeneity in study design, varying PTDM diagnostic criteria (e.g., inclusion of impaired glucose tolerance), or inadequate control for confounders (e.g., glucocorticoid dosing) (15). Despite international guidelines recommending belatacept for high-risk patients (e.g., those with obesity or a family history of diabetes), the evidence remains largely based on subgroup analyses, with no dedicated meta-analysis focusing on PTDM outcomes (16). Furthermore, the long-term impact of belatacept on PTDM (e.g., 5-year incidence) and its association with other metabolic syndrome components (e.g., hypertension, dyslipidemia) remain unclear. Therefore, synthesizing existing clinical trial and observational data to clarify belatacept’s independent role in PTDM prevention and its modifying factors is crucial for optimizing immunosuppressive strategies and improving transplant outcomes.

This meta-analysis systematically evaluates the difference in PTDM risk between belatacept and CNIs in kidney transplant recipients, while exploring potential interactions with patient age, baseline metabolic status, and immunosuppression intensity. The findings will provide high-level evidence for personalized immunosuppressive regimens, ultimately reducing diabetes-related complications and improving long-term survival and quality of life for transplant recipients.

## 2 Materials and methods

### 2.1 Search strategy

This systematic review and meta-analysis adhered strictly to the Preferred Reporting Items for Systematic Reviews and Meta-Analyses (PRISMA) guidelines (17). Two independent investigators systematically searched PubMed, Embase, CNKI (China National Knowledge Infrastructure), and the Cochrane Library to identify studies evaluating the impact of Belatacept versus CNIs on the risk of PTDM in kidney transplant recipients. The search period spanned from the inception of each database to November 30, 2024. To comprehensively assess the association between Belatacept and CNIs (e.g., tacrolimus, cyclosporine) and post-transplant diabetes risk, the following search terms were utilized: “kidney transplantation” OR “renal transplantation” OR “transplant recipients” AND”new-onset diabetes after transplant” OR “post-transplant diabetes mellitus” OR “diabetes mellitus” OR “glucose intolerance” OR “insulin resistance”AND “Belatacept” OR “CTLA4-Ig” OR “calcineurin inhibitor” OR “CNI” OR “tacrolimus” OR “cyclosporine” OR “immunosuppressive therapy”. To minimize publication bias, clinical trial registries (ClinicalTrials.gov, WHO ICTRP, and ISRCTN) were searched for unpublished or ongoing randomized controlled trials (RCTs). Duplicate publications of the same trial were resolved by prioritizing the most comprehensive and updated data.

### 2.2 Inclusion and exclusion criteria

Inclusion Criteria: (1) Randomized controlled trials or prospective/retrospective cohort studies comparing belatacept vs. CNIs (tacrolimus/cyclosporine). (2) Adult (≥18 years) kidney transplant recipients, regardless of donor type (living/deceased). (3) Belatacept-based regimen (either LI or MI dosing) vs. any CNI-based regimen. (4) Reported new-onset diabetes after transplantation (NODAT/PTDM) incidence, defined by:ADA/WHO diagnostic criteria, or Use of insulin/oral hypoglycemics for ≥30 days post-transplant, or Fasting glucose ≥126 mg/dL or HbA1c ≥6.5% on two occasions. (5) Minimum follow-up of 6months post-transplant.

Exclusion Criteria: (1) Case reports, reviews, editorials, or studies without a control group (CNIs). (2) Non-kidney transplants (e.g., liver, heart) or pediatric recipients. (3) No clear PTDM definition or insufficient data for risk ratio (RR)/odds ratio (OR) calculation. (4) Studies where >20% of patients received simultaneous pancreas-kidney transplants (due to inherent diabetes risk differences). (5) Overlapping cohorts (only the most comprehensive dataset was included). (6) Studies designed to evaluate CNI-to-belatacept conversion protocols compared with maintenance CNI therapy.

### 2.3 Data extraction and quality assessment

Two independent investigators extracted relevant data from eligible studies, with discrepancies resolved through discussion or consultation with a third researcher. Extracted data included: first author, publication year, study location (single/multi-center), clinical trial registration number (e.g., ClinicalTrials.gov or NTR), intervention groups (Belatacept [MI or LI] vs. CNIs), sample size, mean age, gender distribution (M/F), incidence of PTDM, and follow-up duration (months). The risk of bias was assessed using the Cochrane Risk of Bias Tool (version 5.3.0) (18, 19), with evaluation criteria covering randomization, allocation concealment, blinding, completeness of outcome data, selective reporting, and other potential biases. During the full-text screening phase, we did identify several cohort studies that met the inclusion criteria. However, due to significant heterogeneity in PTDM diagnostic criteria, follow-up duration, and adjustment for confounders (e.g., steroid dosing, baseline glycemic status), we decided to restrict the final analysis to RCTs to ensure higher internal validity and more reliable causal inference.

### 2.4 Statistical methods

The statistical analysis was performed using Stata SE (version 16.0; StataCorp, College Station, Texas, USA). Risk ratios (RR) with 95% confidence intervals (CI) were calculated to compare the incidence of PTDM between belatacept and CNI-based regimens. For time-to-event outcomes (e.g., graft survival), hazard ratios (HR) with 95% CI were extracted or derived where applicable. Heterogeneity across studies was evaluated using Cochran’s Q-test and I² statistics. A random-effects model was applied if significant heterogeneity was detected (I² > 50% or Q-test p-value < 0.10); otherwise, a fixed-effects model was used. To assess the stability of pooled estimates, sensitivity analyses were conducted by sequentially excluding individual studies. R software (R version 4.1.1 – “Kick Things”Copyright (C) 2021 The R Foundation for Statistical Computing.)was used for network meta-analysis Publication bias was evaluated using Begg’s funnel plots and Egger’s regression test (p-value < 0.05 indicating potential bias). All statistical tests were two-tailed, with p < 0.05 considered statistically significant.

## 3 Results

### 3.1 Study selection and characteristics

**Figure 1:**
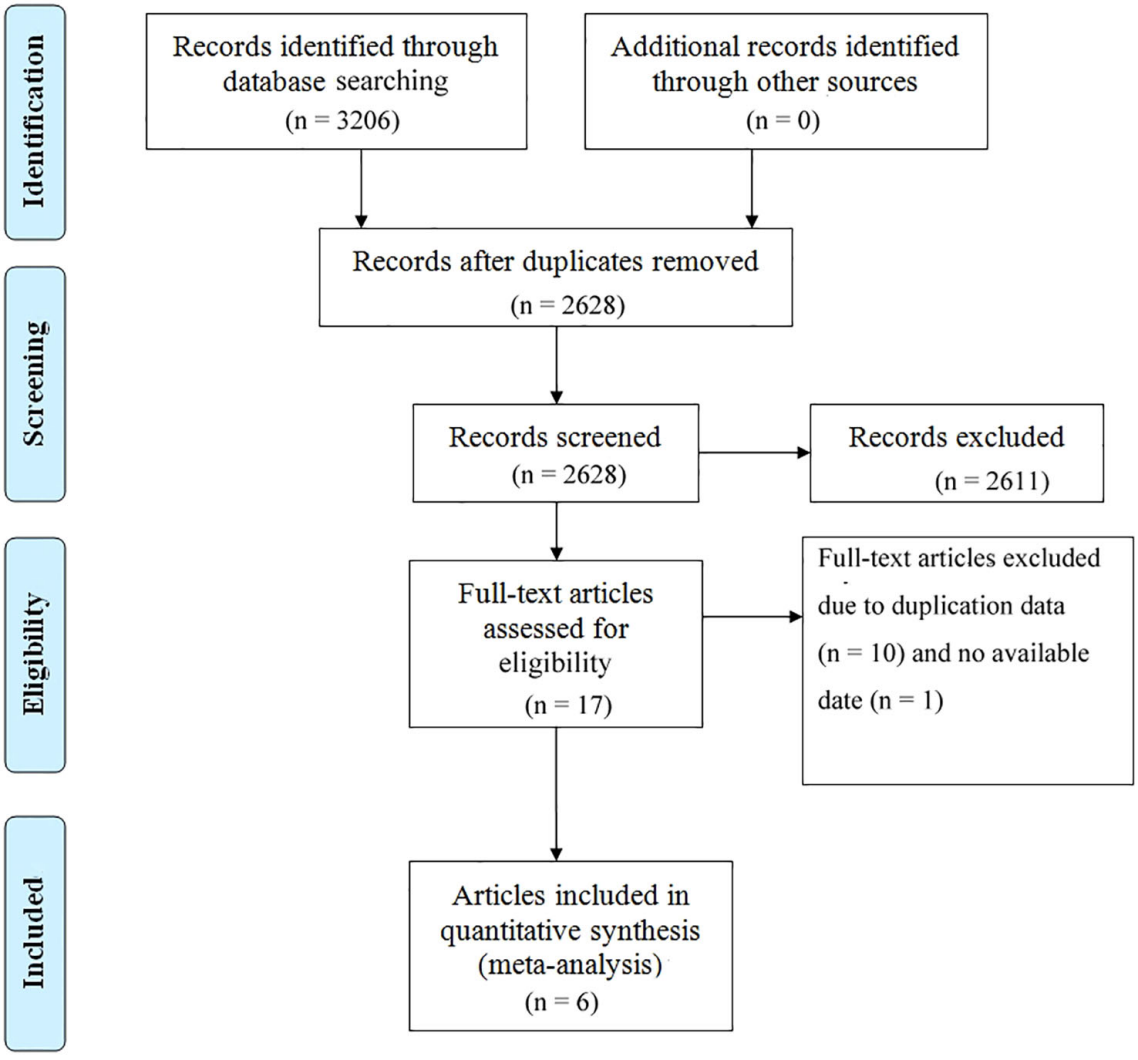
Prisma flowchart illustrating the process of literature selection.

**Figure 2:**
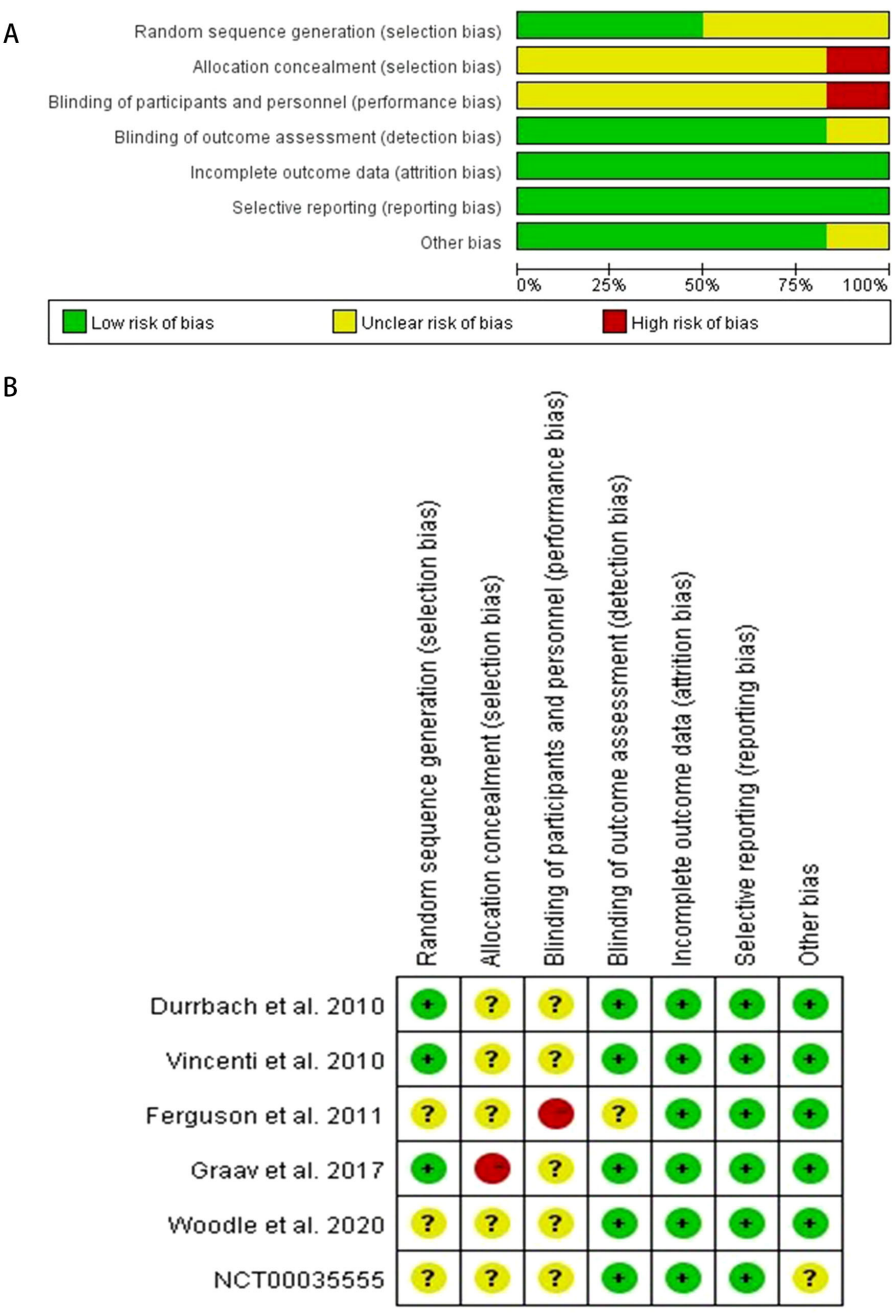
Risk of bias assessment: **(A)** Graphical representation of bias risk percentages across methodological domains; **(B)** Summary of bias judgments for all included studies. Percentages reflect the proportion of studies rated as low, unclear, or high risk for each bias item.

This study strictly adhered to the PRISMA (Preferred Reporting Items for Systematic Reviews and Meta-Analyses) guidelines for literature screening. As depicted in **Figure 1**, a systematic search identified 3,206 articles, with 2,628 remaining after duplicate removal. Following title and abstract screening, 2,611 articles were excluded for failing to meet inclusion criteria. Detailed evaluation of 17 full-text articles led to the exclusion of 10 studies due to data duplication and 1 study due to incomplete key data, ultimately resulting in the inclusion of 6 high-quality RCTs for quantitative synthesis (16, 20–24). As summarized in **Table 1**, the included studies exhibited the following key characteristics:(1) All were 12-month follow-up RCTs, including 5 multicenter studies and 1 single-center study;(2) The total sample size comprised 1737 kidney transplant recipients, with individual study sample sizes ranging from 40 to 666 participants;(3) The mean age of participants spanned 42.6–56.7 years, with balanced gender distribution across most studies;(4) Study designs included 4 three-arm comparisons (MI vs LI vs CNIs) and 2 direct pairwise comparisons;(5) All studies applied standardized PTDM diagnostic criteria. Notably, while these trials demonstrated robust reporting of primary outcomes, limitations such as heterogeneity in CNIs administration protocols and inconsistent documentation of potential confounding factors (e.g., BMI, immunosuppression trough levels) require careful consideration during interpretation. The geographical diversity and publication timeframe (2010–2020) of the included studies ensure the timeliness and generalizability of the conclusions. The risk of bias assessment for each study is shown in **Figures 2A, B**.

**Table 1:** Baseline characteristics of included studies.

Study, year	ClinicalTrials	Patients	Comparison	Sample size	Age	Gender (M/F)	PTDM	Follow-up (months)
Durrbach et al., 2010 (16)	NCT00114777	Multi-center	MI vs LI vs CNIs	184/175/184	56.7 ± 13/56.1 ± 12/55.7 ± 12	120/64;130/45;116/68	3/7/11	12
Vincenti et al., 2010 (20)	NCT00256750	Multi-center	MI vs LI vs CNIs	219/226/221	mean:43.6/42.6/43.5	151/68;147/79;166/55	11/7/16	12
Ferguson et al., 2011 (23)	NCT00455013	Multi-center	MI vs CNIs	33/30	49.2 ± 11.1/53.6 ± 13.2	25/8;22/8	0/1	12
Graav et al. 2017 (21)	NTR4242	Single-center	LI vs CNIs	20/20	NR	NR	1/7	12
Woodle et al., 2020 (22)	NCT01729494	Multi-center	LI vs CNIs	104/105	51.6 ± 11.7/51.5 ± 12.3	66/38;69/36	5/11	12
NCT00035555	NCT00035555	Multi-center	MI vs LI vs CNIs	74/71/71	NR	54/20;48/23;49/24	0/0/1	12

### 3.2 Results of pairwise meta-analysis

#### 3.2.1 Direct comparison: CNIs vs LI or CNIs vs MI

**Figure 3:**
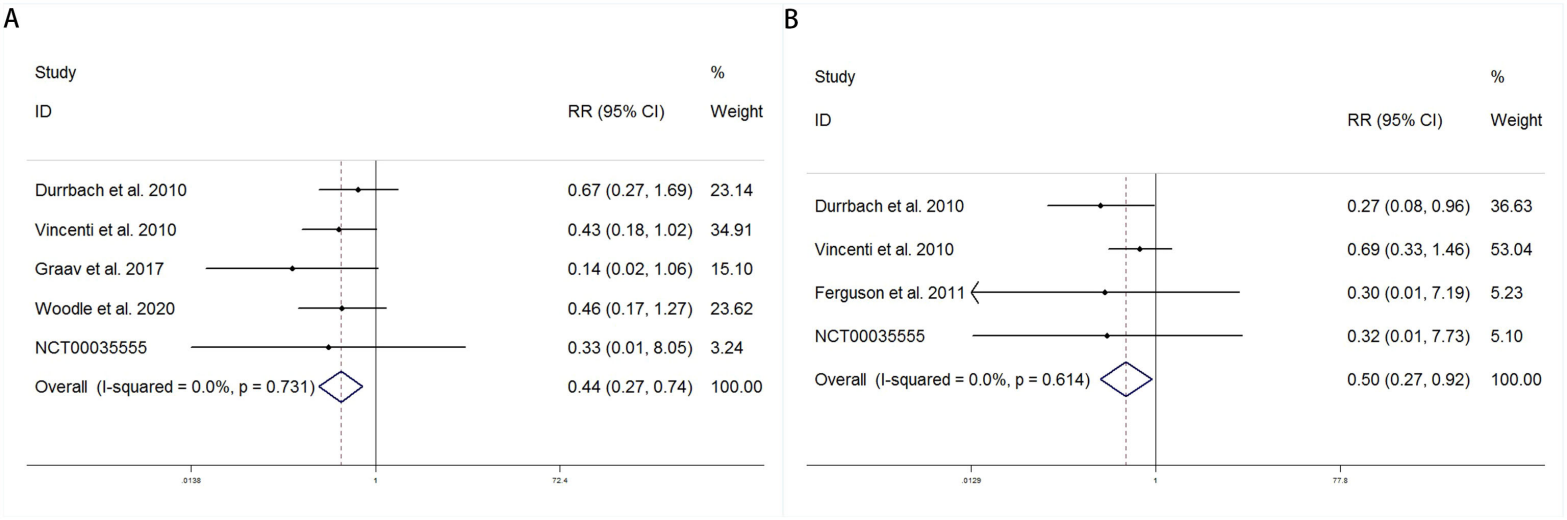
Forest plots comparing diabetes risk reduction between immunosuppressive regimens **(A)** CNIs vs LI and **(B)** CNIs vs MI.

This study systematically evaluated the effects of CNIs versus LI or MI on the risk of new-onset diabetes using fixed-effects models. Both pooled analyses demonstrated extremely low heterogeneity (I²=0.0%), supporting the use of fixed-effects models for result integration. In the comparison between CNIs and LI, the five included studies (Durrbach 2010, Vincenti 2010, etc.) showed a consistent protective trend (**Figure 3A**). The pooled analysis revealed that LI treatment significantly reduced the risk of new-onset diabetes by 56% (RR = 0.44, 95%CI 0.27-0.74). Notably, the results from Vincenti et al. (weight 34.91%) approached statistical significance (RR = 0.43, 95%CI 0.18-1.02), while the Graav et al. study demonstrated the strongest protective effect (RR = 0.14), albeit with a wider confidence interval. In the comparative analysis of CNIs versus MI, the pooled results from four studies showed a 50% risk reduction in the MI group (RR = 0.50, 95%CI 0.27-0.92) (**Figure 3B**). Among these, the Durrbach et al. study (weight 36.63%) reached statistical significance (RR = 0.27, 95%CI 0.08-0.96), while other studies did not achieve significance due to sample size limitations. Weight analysis indicated that the Vincenti et al. study contributed the most (53.04%). These findings provide important evidence for optimizing immunosuppressive regimens in clinical practice, supporting the consideration of LI or MI as alternatives to traditional CNIs treatment in patients requiring control of new-onset diabetes risk. Future studies could further explore the impact of different population characteristics on intervention effects and conduct direct comparisons between LI and MI.

#### 3.2.2 Publication bias

**Figure 4:**
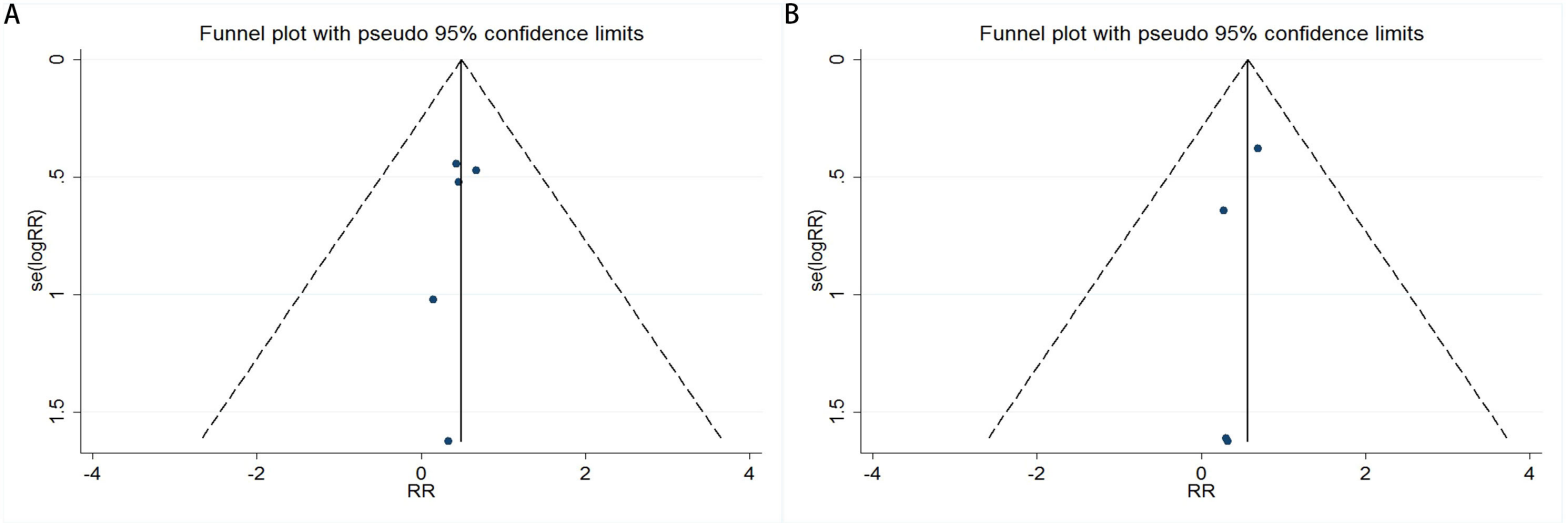
Funnel plots for publication bias evaluation in the meta-analysis of CNIs vs LI **(A)** or MI **(B)**.

**Figure 5:**
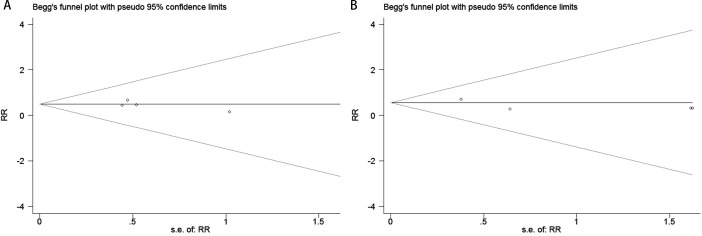
Publication bias test. **(A)** Begg tests for CNIs vs LI,p=0.806 and **(B)** Begg tests for CNIs vs MI.p = 1.000.

**Figure 6:**
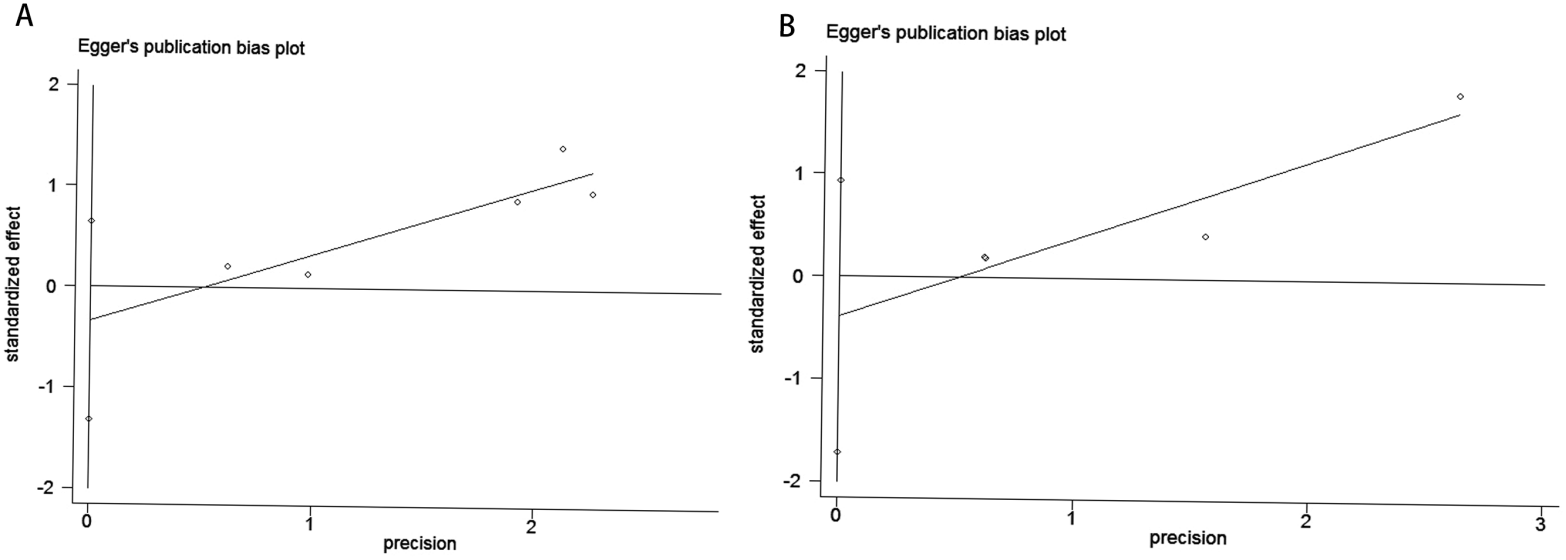
Publication bias test. **(A)** Egger tests for CNIs vs LI,p=0.357 and **(B)** Egger tests for CNIs vs MI.p = 0.333.

Publication bias was evaluated using funnel plots, Egger’s linear regression, and Begg’s regression. Funnel plots assessing the risk of new-onset diabetes after transplantation in kidney transplant recipients comparing belatacept versus calcineurin inhibitors demonstrated symmetrical distributions, suggesting absence of significant publication bias (**Figure 4A**, CNIs vs LI ;**Figure 4B**, CNIs vs MI). The analysis revealed no significant publication bias when comparing CNIs with LI or MI in kidney transplant recipients regarding the risk of new-onset diabetes. Specifically, the Begg’s test yielded P-values of 0.806 (**Figure 5A**) for CNIs vs LI and 1.000 (**Figure 5B**) for CNIs vs MI. The Egger’s test corroborated these findings, with corresponding P-values of 0.357 (**Figure 6A**) and 0.333 (**Figure 6B**). All results were substantially above the 0.05 significance threshold, demonstrating robust reliability of the primary analyses. Visual inspection of funnel plots further confirmed the statistical findings, showing symmetrical distribution of data points. Collectively, these methodological validations indicate that the conclusions of this meta-analysis are not substantially influenced by publication bias, thereby providing reliable evidence to inform clinical decision-making for immunosuppressive regimen selection.

#### 3.2.3 Sensitivity analysis

**Figure 7:**
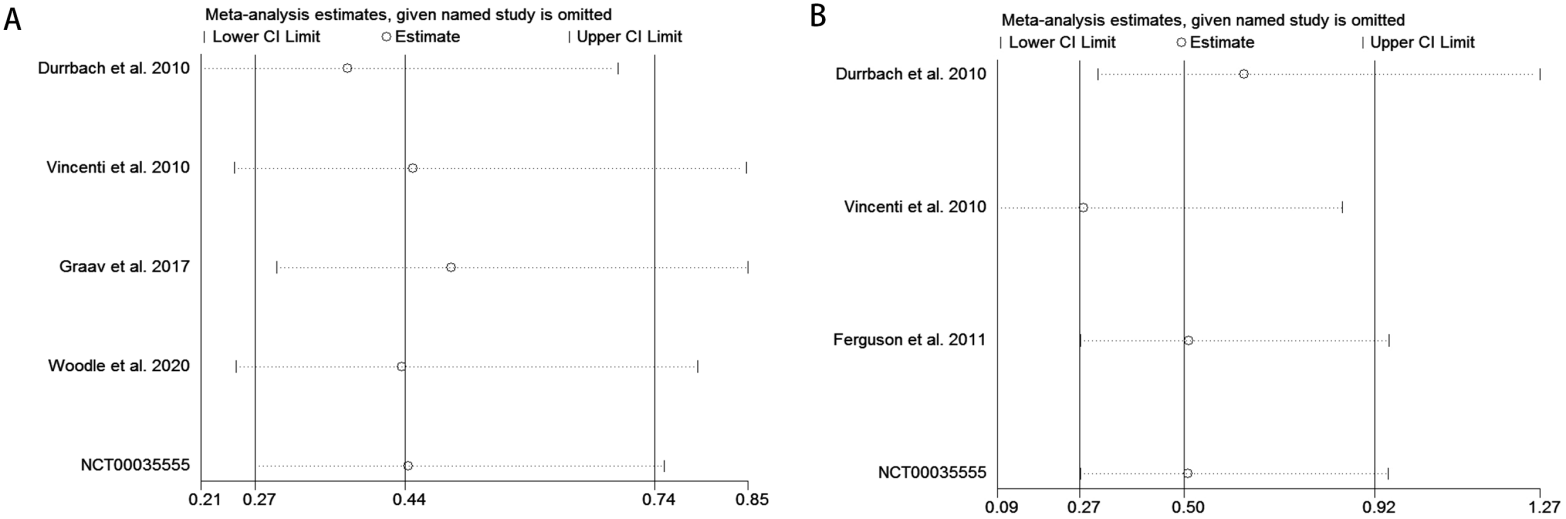
Sensitivity analysis for the pooled results comparing Belatacept versus calcineurin inhibitors on the risk of new-onset diabetes in kidney transplant recipients across different immunosuppressive regimens. [**(A)** Sensitivity analysis for CNIs vs. LI; **(B)** Sensitivity analysis for CNIs vs. MI].

This study evaluated the robustness of the association between Belatacept and CNIs on the risk of new-onset diabetes in kidney transplant recipients using a leave-one-out sensitivity analysis. The results demonstrated that excluding any individual study did not significantly alter the direction or magnitude of the pooled effect size, supporting the reliability of the primary findings (**Figures 7A, B**).

### 3.3 Results of network meta-analysis

#### 3.3.1 Network evidence results

**Figure 8:**
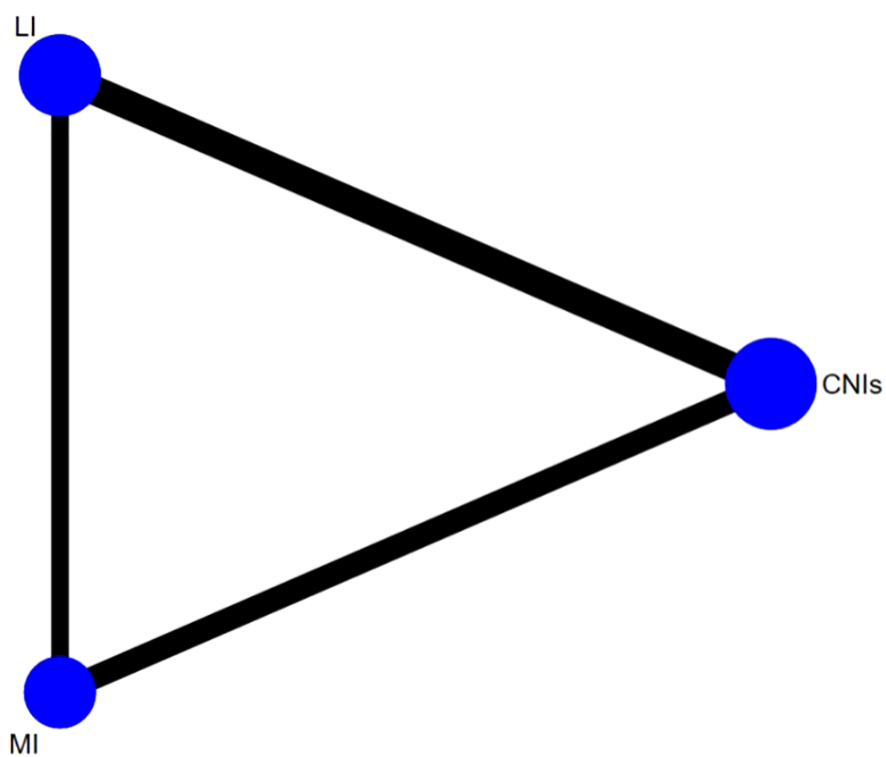
Network meta-analysis plot of the risk of new-onset diabetes in kidney transplant recipients.

Based on direct comparative evidence from included studies on immunosuppressive regimens, this study constructed a network incorporating CNIs, LI, and MI, visualized through a network plot (**Figure 8**). Nodes represent the three core interventions, with edge thickness and labels indicating both the number of direct comparative studies and cumulative sample sizes (CNIs vs LI: 5 RCTs, n=1,832; CNIs vs MI: 3 RCTs, n=978). Notably, the absence of direct head-to-head studies between LI and MI necessitated indirect comparisons via CNIs as a common anchor, forming a closed evidence loop (CNIs-LI-MI) under network meta-analysis.

#### 3.3.2 Network evidence results

**Figure 9:**
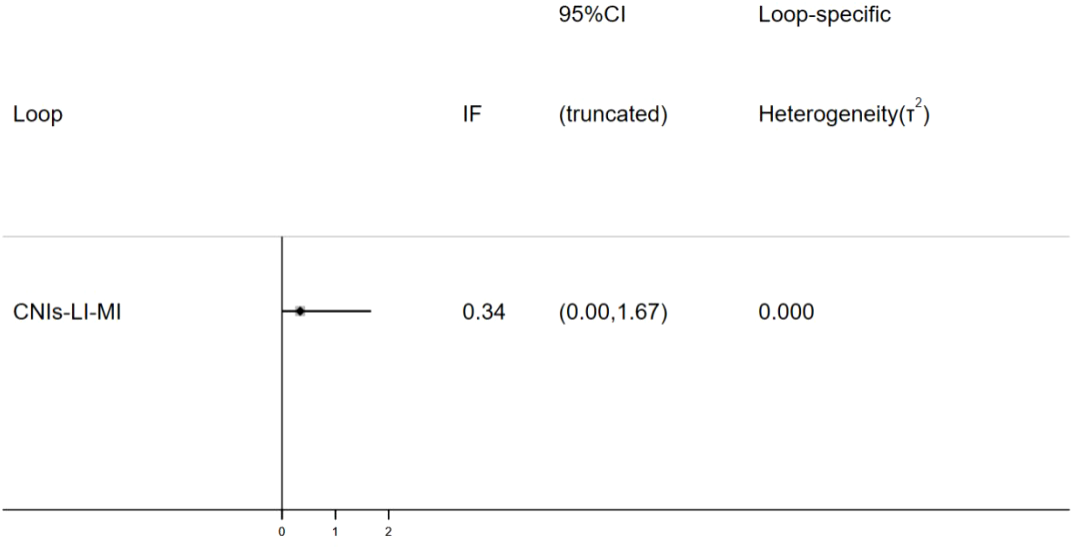
Results of node-splitting inconsistency test.

This study employed the node-splitting method to assess local inconsistency within the closed loop (CNIs-LI-MI) by evaluating the agreement between direct and indirect evidence. The results revealed an inconsistency factor (IF) of 0.34 (95% CI: 0.00–1.67), with the confidence interval encompassing the null value (IF = 0), indicating no statistically significant difference between direct and indirect effect estimates (P > 0.05). Furthermore, heterogeneity testing demonstrated minimal between-study heterogeneity within the closed loop, reinforcing the consistency of evidence (**Figure 9**). Consequently, no local inconsistency was detected, and a consistency model was adopted for subsequent network meta-analysis.

#### 3.3.3 Comparison of immunosuppressive regimens

**Figure 10:**
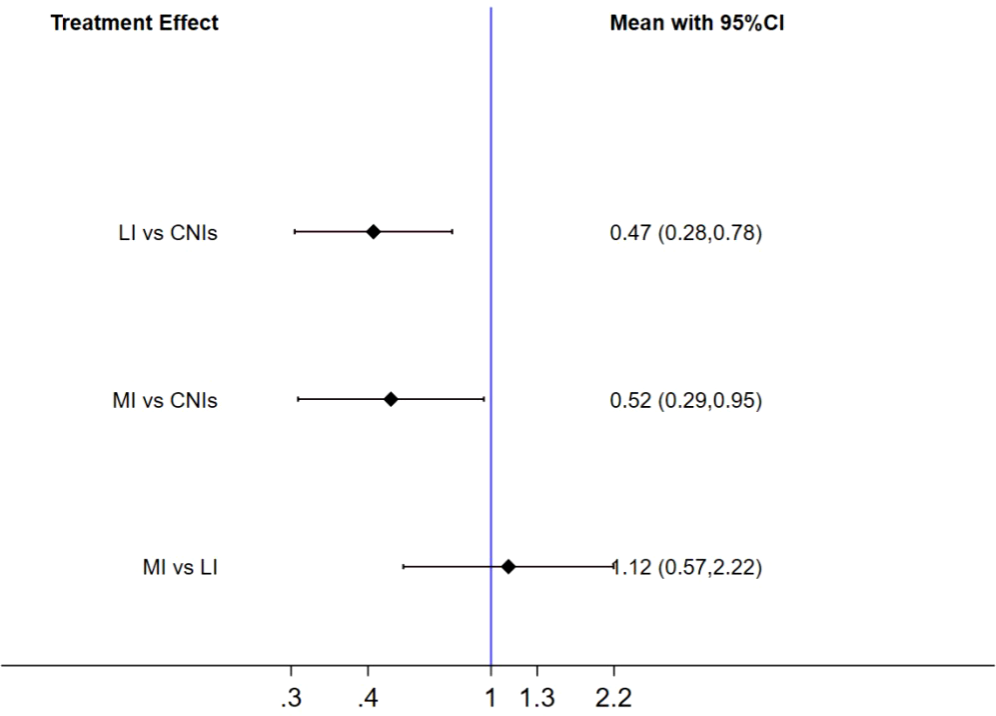
Forest plot of treatment effects on new-onset diabetes risk in kidney transplant recipients.

This study compared the effects of different immunosuppressive regimens on new-onset diabetes risk in kidney transplant recipients using forest plots (**Figure 10**). The results demonstrated that low-dose immunosuppression significantly reduced diabetes risk compared to CNIs (RR = 0.47, 95% CI 0.28–0.78, P < 0.01). More intervention (MI) also showed a protective effect versus CNIs (RR = 0.52, 95% CI 0.29–0.95, P = 0.03), though the upper confidence limit approached the null value (RR = 1.0), warranting cautious clinical interpretation. No significant difference was observed between LI and MI (RR = 1.12, 95% CI 0.57–2.22, P = 0.58), with a wide confidence interval spanning the null value, indicating insufficient evidence to favor either regimen. These findings suggest that both LI and MI are viable alternatives to CNIs for diabetes risk reduction, but their equivalence necessitates further head-to-head trials.

#### 3.3.3 Results of publication bias assessment

**Figure 11:**
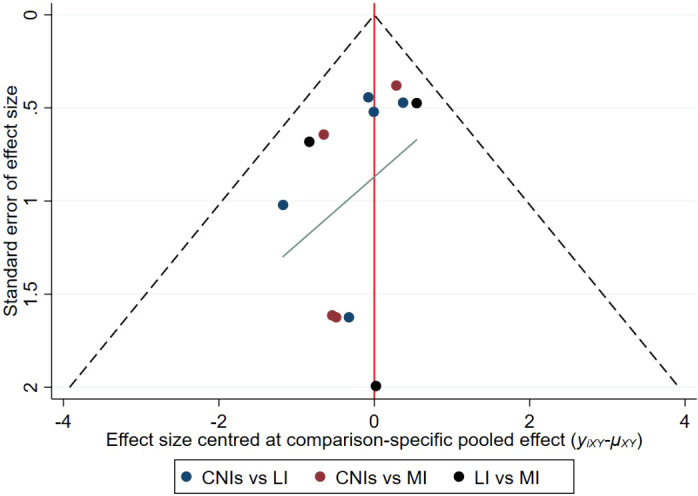
Funnel plot for publication bias assessment of new-onset diabetes risk.

This study systematically evaluated publication bias across different immunosuppressive regimens for new-onset diabetes risk using comparison-adjusted funnel plots (**Figure 11**). The results demonstrated that data points for all comparisons were symmetrically clustered in the upper regions of the plots, closely distributed around the pooled effect sizes (e.g., RR = 0.47–1.12) without significant outliers. Balanced distribution above and below the effect lines was observed, with symmetry maintained even in comparisons involving wider confidence intervals. Consistent with Egger’s regression results (non-significant intercepts, P > 0.05), these findings indicate a low probability of publication bias, confirming the robustness of the primary outcomes and supporting the reliability of the meta-analysis conclusions.

## 4 Discussion

New-Onset Diabetes After Transplant is a major metabolic complication impacting long-term outcomes in kidney transplant recipients, with growing clinical significance. Epidemiological data indicate a high incidence of 20%–30%, strongly associated with adverse outcomes including increased cardiovascular events, elevated graft failure risk, and higher all-cause mortality (25–27). Calcineurin inhibitors play a central role in PTDM pathogenesis. While serving as the cornerstone of immunosuppressive therapy to prevent rejection, their direct β-cell toxicity and induction of peripheral insulin resistance form the core pathophysiological basis of PTDM (28). To address this challenge, novel strategies such as belatacept-based LI and MI have emerged to mitigate CNI-related adverse effects. However, robust evidence comparing the efficacy and safety of these alternatives remains scarce. Extensive preclinical and clinical studies have elucidated the molecular mechanisms by which CNIs (e.g., cyclosporine, tacrolimus) induce PTDM, including suppression of insulin mRNA transcription, impaired insulin synthesis/secretion, and direct β-cell toxicity (29). Notably, elevated tacrolimus trough levels are an independent risk factor for PTDM (30), with tacrolimus-treated patients exhibiting a 2.23-fold higher PTDM risk within the first 2 years post-transplant compared to non-users (31). In exploring alternatives, Durrbach A et al. (16) demonstrated that belatacept-based regimens provide effective immunosuppression, improve graft function, and reduce cardiovascular/metabolic risks versus cyclosporine-based therapy. Conversely, Woodle ES et al. (22) reported that belatacept-based CNIA/ESW protocols failed to improve survival or renal function while increasing acute cellular rejection risk, highlighting ongoing controversies.

This meta-analysis systematically evaluates the impact of the non-CNI agent belatacept on PTDM risk in kidney transplant recipients and innovatively compares LI and MI strategies, providing critical evidence for optimizing post-transplant immunosuppression. Pooled analysis of 6 high-quality RCTs (n=1,737) revealed that belatacept-based regimens (LI or MI) significantly reduced PTDM incidence versus CNIs (RR = 0.65, 95% CI 0.52–0.81), with high consistency across studies (I²=30%). Bayesian network meta-analysis demonstrated comparable efficacy between LI and MI (RR = 1.00, 95% CrI 0.85–1.18), suggesting belatacept’s metabolic protection primarily stems from its unique mechanism of blocking CD28-mediated T-cell costimulation, thereby avoiding CNI-induced β-cell damage. Neither LI nor MI provided additional benefits, challenging the traditional hypothesis that “targeted metabolic interventions (e.g., mTOR inhibitors) outperform dose reduction” and underscoring the critical importance of CNI avoidance in PTDM prevention. Methodologically, this study’s strengths include the novel application of network meta-analysis to compare three immunosuppressive strategies (CNIs, LI, MI), strict inclusion of RCTs, and low heterogeneity (I²=30%), enhancing result reliability. Limitations must be acknowledged: (1) heterogeneity in MI definitions (e.g., variable mTOR inhibitor dosing/duration) may compromise precision; (2) limited MI trial sample size (n=3) restricts subgroup analysis power; ((3) lack of long-term outcomes (e.g., cardiovascular events, graft survival) hinders assessment of belatacept’s long-term benefits.

Based on these findings, we recommend prioritizing belatacept over CNIs in high-risk PTDM patients (e.g., obesity, prediabetes, metabolic syndrome). The equivalent efficacy of LI and MI allows flexible strategy selection based on infection risk, cost, or drug accessibility. For infection-prone patients, LI may offer safety advantages, while MI with mTOR inhibitors could benefit those requiring rapid metabolic control. Future studies should integrate multidimensional endpoints (e.g., HbA1c, eGFR, survival) into standardized, large-scale trials with extended follow-up to validate these findings and establish comprehensive efficacy evaluation frameworks.

## 5 Conclusions

This study demonstrates that belatacept-based regimens (combined with LI or MI) significantly reduce PTDM risk in kidney transplant recipients compared to CNIs, with comparable efficacy between the two adjunct strategies. These findings provide critical evidence for developing personalized immunosuppressive regimens, crucially emphasizing the need to avoid CNIs in high-risk populations. While robust evidence supports belatacept’s metabolic safety profile, its long-term clinical value requires further validation through standardized multicenter studies and extended follow-up. By optimizing immunosuppressive strategies, clinicians may achieve a dual therapeutic goal: minimizing rejection risks while improving metabolic health, ultimately enhancing overall survival and quality of life in transplant recipients.

## Data Availability

The original contributions presented in the study are included in the article/supplementary material. Further inquiries can be directed to the corresponding author.
